# Genetic Ablation of Na,K-ATPase α4 Results in Sperm Energetic Defects

**DOI:** 10.3389/fcell.2022.911056

**Published:** 2022-05-26

**Authors:** September Numata, Jeffrey P. McDermott, Gustavo Blanco

**Affiliations:** Department of Molecular and Integrative Physiology, University of Kansas Medical Center, Kansas City, KS, United States

**Keywords:** sperm energetics, glycolysis, mitochondria, Na,K-ATPase, NAD/NADH

## Abstract

The Na,K-ATPase alpha 4 isoform (NKAα4) is expressed specifically in the male germ cells of the testes and is particularly abundant in mature spermatozoa. Genetic deletion of NKAα4 in mice (NKAα4 KO mice) results in complete infertility of male, but not female mice. The reduced fecundity of NKAα4 KO male mice is due to a series of defects, including a severe impairment in total and hyperactive sperm motility. In this work, we show that deletion of NKAα4 also leads to major defects in sperm metabolism and energetics. Thus, compared to wild-type sperm, sperm from NKAα4 KO mice display a significant reduction in the extracellular acidification rate (ECAR), indicative of impaired glycolytic flux. In addition, mitochondrial function is disrupted in sperm lacking NKAα4, as indicated by a reduction in the mitochondrial membrane potential and lower oxygen consumption rate (OCR). Moreover, the ratio between the oxidized and reduced forms of nicotinamide adenine dinucleotide (NAD/NADH) is increased in NKAα4 KO sperm, indicating a shift in the cellular redox state. These metabolic changes are associated with augmented reactive oxygen species (ROS) production and increased lipid peroxidation in NKAα4 KO sperm. Altogether, these findings reveal a novel link between NKAα4 activity and sperm energetics, highlighting the essential role of this ion transporter in sperm physiology.

## Introduction

As sperm travel through the male and female reproductive tracts, their function is regulated by many factors within the extracellular fluids, including metabolites, proteins, and ions. The fluctuations in ion concentration are essential for the regulation of sperm motility and fertility. Intracellular ion levels are tightly controlled by a series of ion transport systems expressed at the sperm plasma membrane. Interestingly, a considerable number of these ion translocators are molecular variants of homologous proteins expressed in somatic cells (Navarro et al., 2008; Visconti et al., 2011; Lishko et al., 2012; Wang et al., 2021). Among them is a testis-specific isoform of the Na,K-ATPase (NKA) (Blanco et al., 1999; McDermott et al., 2012).

NKA is a primary transport system that utilizes the free energy from ATP hydrolysis to maintain the low intracellular Na^+^ and high cytosolic K^+^ concentration typical of most animal cells. The uneven Na^+^ and K^+^ distribution generated by NKA is important for maintaining ionic homeostasis, resting membrane potential, and the secondary transport of other ions, solutes, and water across the cell surface (Jorgensen et al., 2003; Clausen et al., 2017).

In mammals, NKA is expressed as an oligomer of two main subunits known as the α and β polypeptides. The α subunit is the catalytic portion of the NKA, responsible for the translocation of Na^+^ and K^+^ across the plasma membrane. The β polypeptide aids in the proper folding and targeting of the α subunit to the plasma membrane (Geering, 1991). Both the NKA α and β polypeptides are expressed as multiple isoforms (designated NKAα1, α2, α3, α4, β1, β2, and β3), which present a tissue specific and developmentally regulated pattern of expression (Blanco, 2005). Sperm express two isoforms of NKA, the ubiquitous NKAα1 isoform, and the testis-specific NKAα4 isoform. NKAα4 and NKAα1 exhibit different affinities for Na^+^, K^+^, and ATP that may better adapt NKA function to the unique requirements of spermatozoa (Blanco et al., 1999; McDermott et al., 2012). NKAα4 is localized mainly to the midpiece of the mouse sperm flagellum, where it is important for motility under non-capacitating conditions, as well as for the hyperactivated pattern of motility observed during sperm capacitation (Jimenez et al., 2011).

Genetic deletion of NKAα4 results in complete infertility of male, but not female mice. Sperm from NKAα4 knockout mice display a severe reduction in all patterns of motility and are unable to fertilize oocytes *in vitro*. The reduced functionality of NKAα4 KO sperm is due to a series of defects, including a disruption of the transmembrane Na^+^ gradient, depolarization of the plasma membrane, low cytoplasmic pH, and high calcium levels (Jimenez et al., 2011).

In this work, we demonstrate that genetic ablation of NKAα4 negatively impacts energy producing pathways in mouse sperm, including glycolysis and mitochondrial oxidative phosphorylation (OXPHOS). This is associated with a shift in the sperm redox status, increased production of reactive oxygen species (ROS), and lipid peroxidation. Altogether, these energetic defects have been shown to be deleterious to sperm function and contribute to the infertility observed in male NKAα4 KO mice (Miki et al., 2004; Odet et al., 2008; Danshina et al., 2010; Nakamura et al., 2013; Ferramosca and Zara, 2014; Agnihotri et al., 2016; Nowicka-Bauer and Nixon, 2020).

## Materials and Methods

### Sperm Preparation

The design and engineering of the NKAα4 KO mouse has been published elsewhere (Jimenez et al., 2011). Spermatozoa from 12–18 week-old wild type (WT) and NKAα4 KO male mice were obtained from the cauda epididymides after swim-up of the cells, as previously described (Jimenez et al., 2011). Sperm were resuspended in modified Whitten’s medium, containing: 100 mM NaCl, 4.7 mM KCl, 1.2 mM KH_2_PO_4_, 1.2 mM MgSO_4_, 1.7 mM CaCl_2,_ 5.5 mM Glucose, 0.8 mM pyruvic acid, 4.8 mM lactic acid, 20 mM Hepes (pH 7.4). Cells were counted and used for the different assays. For some experiments, sperm were capacitated in the medium described above, supplemented with 25 mM sodium bicarbonate and 0.5% BSA. All experimental protocols involving animals in this work were approved by the University of Kansas Medical Center Institutional Animal Care and Use Committee.

### Analysis of Sperm Energetics Using Seahorse XF

The extracellular acidification rate (ECAR) and oxygen consumption rate (OCR) were measured using the Seahorse XF Analyzer (Agilent, Santa Clara, CA). Approximately 72 h before the assay, a 24 well plate was coated with 45 ul of Concanavalin A (0.5 mg/ml) and allowed to air dry. The night before the assay, the sensor cartridge was loaded with 200 µl of calibrant and placed in a 37°C incubator. The day of the experiment, mice were euthanized, and sperm were swam up in non-capacitated media containing 120 mM NaCl, 4.7 mM KCl, 1.7 mM CaCl_2_, 1.2 mM KH_2_PO_4_, 1.2 mM MgSO_4_, and 5.5 mM glucose. The sensor cartridge was loaded with the corresponding compounds. Port A was loaded with carbonyl cyanide 4-(trifluoromethoxy)phenylhydrazone (FCCP) at a final concentration 0.1 μM, Port B with Antimycin A/Rotenone (AA + R) (final concentration 0.1 μM), and Port C with 2-Deoxyglucose (2-DG) (final concentration 1 mM). After sperm were swam up for approximately 20 min, cells were washed twice and resuspended in media. Cells were seeded at 2 × 10^6^ sperm per well on a 24-well plate. For each assay, the values of three wells were averaged for each group (WT or NKAα4 KO) and at least four wells were reserved for media alone to correct for background. After calibration with the sensor cartridge, the cell plate was loaded into the Seahorse Analyzer. Experiments were repeated 3 times and the data were combined and analyzed using Seahorse Wave Controller Software 2.4 from Agilent (Santa Clara, CA) and statistical analysis was performed using Graph Pad Prism (San Diego, CA).

### Determination of Mitochondrial Membrane Potential

The transmembrane voltage difference of sperm mitochondria was measured using the potentiometric dye, tetraethylbenzimidazolylcarbocyanine iodide (JC-1) (Thermo fisher, Waltham, MA). Briefly, mice were sacrificed, and sperm were swam up in Whitten’s media. Cells were washed once with PBS and resuspended in either Whitten’s or capacitating media. Five hundred µl (1 × 10^6^) of the sperm suspension was added to each tube. To each tube, 10 µL of 200 µM JC-1 was added and incubated at 37°C for 30 min. Cells were washed, resuspended in PBS, and analyzed on a flow cytometer (BD LSR II Flow Cytometer, BD Biosciences, Franklin NJ), with 488 and 633 nm excitation. The data is represented as the percentage of cells with red/green fluorescence over the total number of cells.

### Determination of NAD/NADH

The ratio of NAD:NADH was measured using the NAD/NADH Assay Kit (Abcam, ab65348). Sperm were collected in Whitten’s Media and washed twice with PBS. The pellet was resuspended in extraction buffer, homogenized, and sonicated. The cells were then centrifuged at 10,000 × g for 3 min at 4°C. The supernatant was collected and used for the assay, following the instructions provided by the manufacturer. After samples were allowed to develop for 1 h at room temperature, absorbance was measured at 450 nm using the Flex Station 3 (Molecular Devices, San Jose, CA). Data is presented as the ratio of NAD/NADH normalized to the WT values for each experiment.

### ATP Level Measurement

The levels of ATP in sperm from WT and NKAα4 KO mice were determined using the ATP Determination KIT (A22066, Thermofisher, Waltham, MA), as described previously (Numata et al., 2022). ATP was measured in non-capacitated sperm and in sperm capacitated for 30 and 90 min. Measurements were made using a BioTek Synergy HT plate reader (Bio-Tek, Winooski, VT). ATP levels were normalized to pmol/10^6^ cells.

### Determination of Reactive Oxygen Species

ROS was determined using the Cellular Reactive Oxygen Species Detection Assay Kit (ab186027, Abcam, Waltham, MA). Sperm were collected in Whitten’s Media, washed, and resuspended at 5 × 10^5^ cells per 100 μL. Sperm were added to a 96-well plate in triplicates and treated with ROS Red Stain for 1 h at 37°C. Fluorescence was measured using the Flex Station 3. For capacitated samples, sperm were incubated at 37°C in 5% CO_2_ before adding the ROS Red Stain. The data is quantified as Relative Fluorescent Units (RFU).

### Determination of Lipid Peroxidation

The peroxidation of lipids was determined by immunoblot and immunocytochemistry, using an antibody generated against protein adducts of 4-HNE (ab46545, Abcam, Waltham, MA). Briefly, samples from WT and NKAα4 KO mice were homogenized in RIPA buffer and centrifuged as described previously (Numata et al., 2022). Proteins were separated in a 10% SDS-PAGE gel and transferred onto a PVDF membrane, where they were probed with the anti-4-HNE antibody. Horseradish peroxidase-conjugated secondary antibodies (Jackson ImmunoResearch Laboratories, West Grove, PA) and chemiluminescence were used for detection. Membranes were then washed and stained with Coomassie Blue for normalization to total protein levels. Immunocytochemistry for 4-HNE in WT and NKAα4 KO sperm was performed as described previously (McDermott et al., 2021). Sperm were collected from the epididymis and fixed in 4% PFA, washed, and dropped onto a clean slide. Cells were permeabilized with 0.3% Triton X-100, blocked with 5% BSA, and probed with anti-4-HNE overnight. The next day, slides were then washed and probed with Alexa-Fluorophore 594. DAPI was used as a nuclear counterstain.

### Statistical Analysis

Experiments were repeated at least three times. Statistical significance of differences between samples was determined by the Student’s *t*-test, using GraphPad Prism (San Diego, CA). Statistical significance was defined as *p* < 0.05.

## Results

### Deletion of NKAα4 Impairs Sperm Glycolysis

**FIGURE 1:**
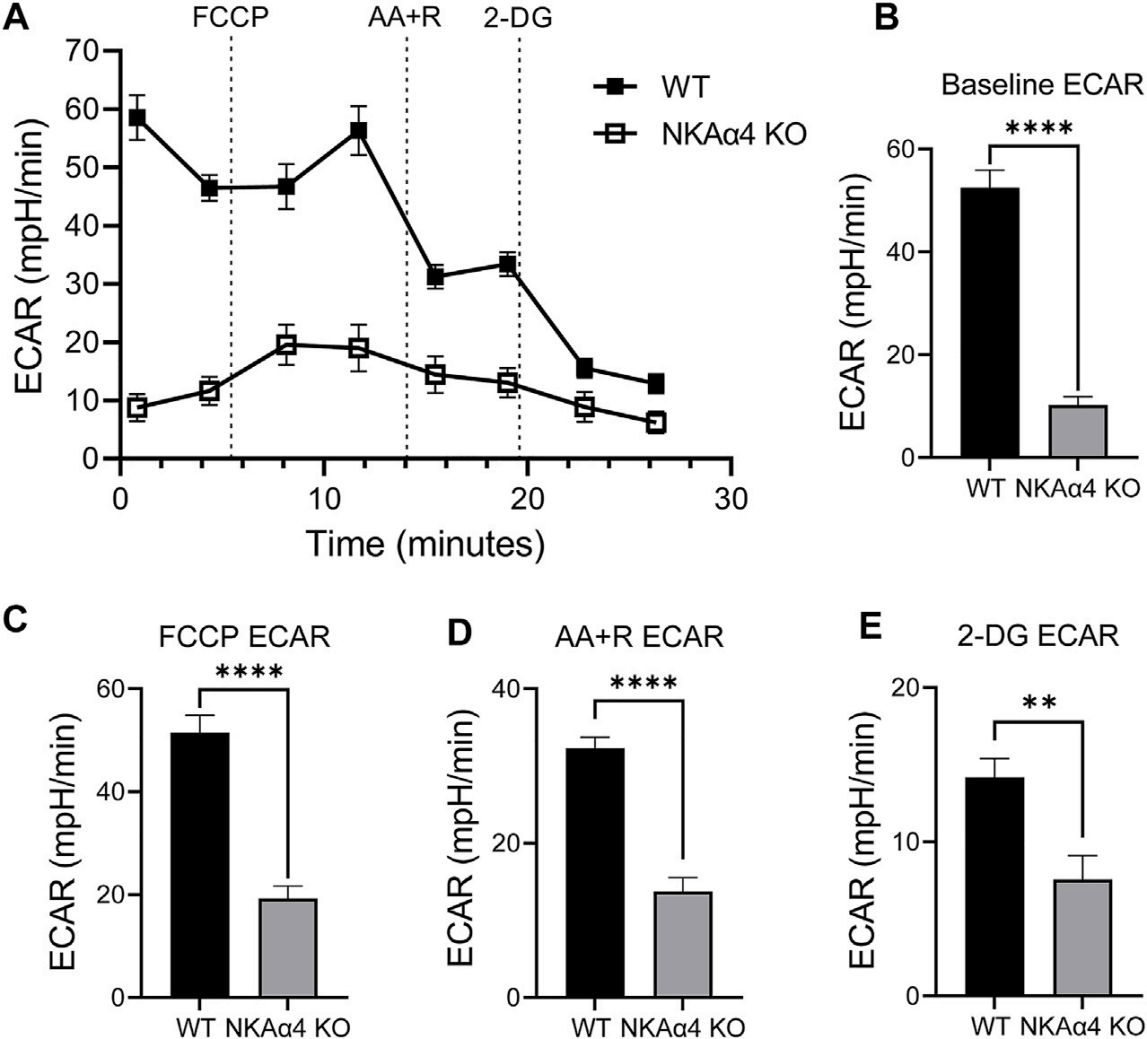
Sperm from NKAα4 KO mice display a significant reduction in the extracellular acidification rate (ECAR) compared to WT sperm. **(A)** ECAR was measured in non-capacitated WT and NKAα4 KO sperm using a Seahorse XF Analyzer after subsequent injections of FCCP, Antimycin A/Rotenone, and 2-Deoxyglucose. Results are expressed as mpH/min released from the sperm, indicative of glycolytic activity. **(B)** Basal ECAR levels. **(C)** ECAR after addition of FCCP. **(D)** Effect of AA + R on ECAR. **(E)** ECAR in the presence of 2-Deoxyglucose. The data are expressed as the mean ± SEM of three different experiments. Asterisks indicate significant differences, with ∗∗*p* < 0.01 and ∗∗∗∗*p* < 0.0001.

Previous observations in our lab have shown that sperm from NKAα4 KO mice have decreased ATP levels compared to WT sperm (Numata et al., 2022). This suggested that NKAα4 deletion may be associated with abnormal energy production. To explore this, we assessed the activity of the two primary energy producing pathways in sperm, glycolysis and oxidative phosphorylation. Glycolytic activity was assessed by measuring the extracellular acidification rate (ECAR), using a Seahorse XF Analyzer. As shown in Figure 1A, sperm from NKAα4 KO mice displayed a significant reduction in ECAR compared to WT sperm. The differences between WT and NKAα4 KO sperm samples were quantified by averaging the values for the basal ECAR, as well as the ECAR under the different pharmacological manipulations performed (Figures 1B–E). The basal glycolytic activity in sperm from NKAα4 KO mice was severely reduced and approximately 5-fold lower than WT sperm glycolytic activity (Figure 1B). Addition of the mitochondrial uncoupler, FCCP, produced the expected increase in ECAR in WT sperm, which was less pronounced for the NKAα4 KO sperm (Figure 1C). The electron transport chain inhibitors, AA + R, only had an effect on WT sperm; however, the ECAR values remained significantly higher in WT than NKAα4 KO sperm (Figure 1D). Finally, treatment with the non-hydrolysable substrate, 2-Deoxyglucose (2-DG), reduced glycolytic activity in WT sperm to levels similar to NKAα4 KO sperm (Figure 1E). Altogether, these data demonstrate that glycolytic activity is severely impaired in sperm lacking NKAα4.

### Sperm Devoid of NKAα4 Exhibit Abnormal Mitochondrial Function

**FIGURE 2:**
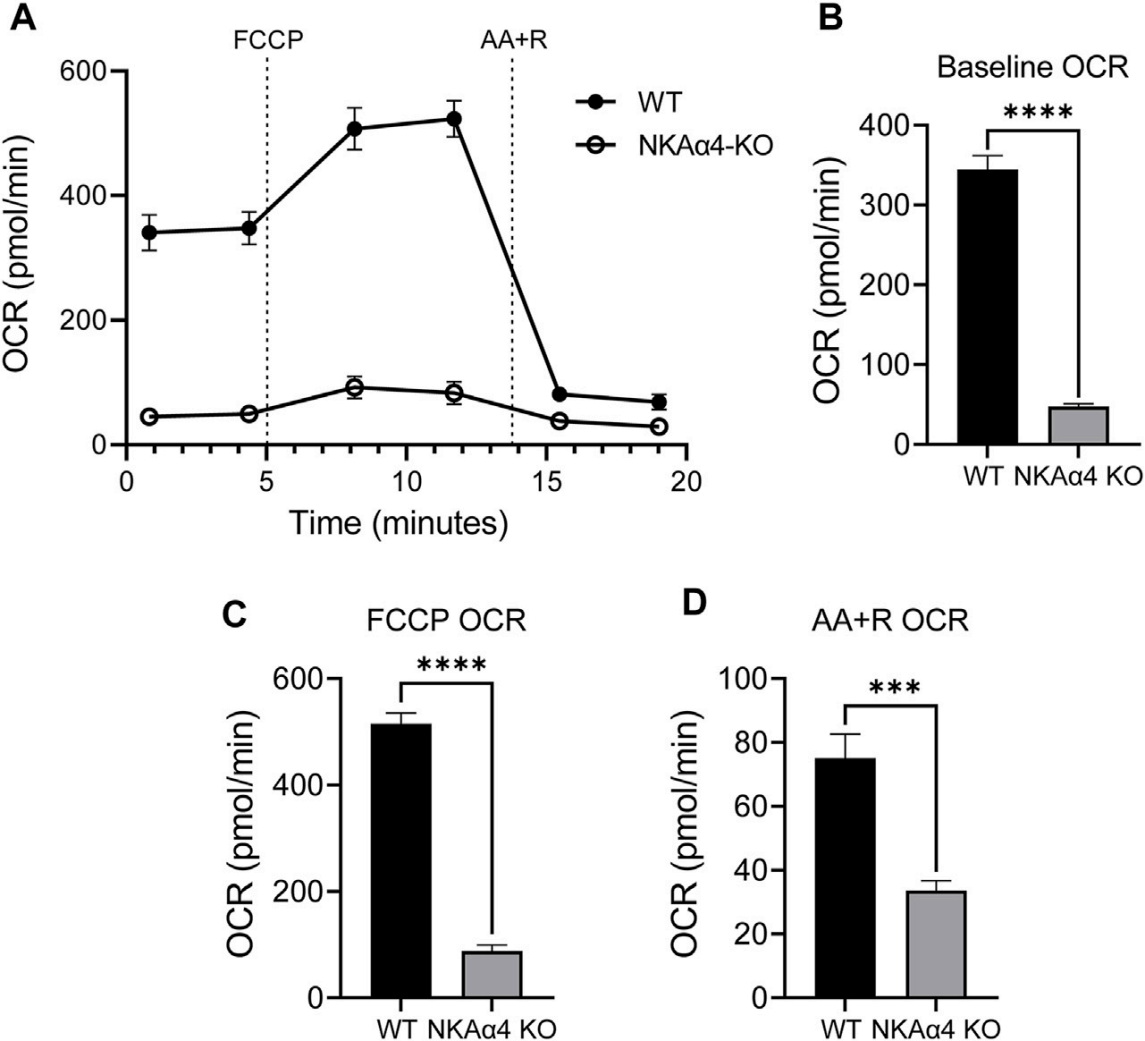
NKAα4 KO sperm exhibit a substantial decrease in the oxygen consumption rate (OCR) compared to WT sperm. **(A)** Oxygen consumption was measured in non-capacitated sperm from NKAα4 KO and WT mice in pmol/min as measured by the Seahorse Analyzer. Sperm were subjected to injections of FCCP, Antimycin A/Rotenone, and 2-Deoxyglucose. **(B)** Basal OCR levels. **(C)** OCR after addition of FCCP. **(D)** Effect of AA + R on OCR. The data are expressed as the mean ± SEM of three different experiments. Asterisks indicate significant differences, with ∗∗∗*p* < 0.001 and ∗∗∗∗*p* < 0.0001.

To explore the effect of NKAα4 deletion on mitochondrial activity in sperm, we measured the oxygen consumption rate (OCR) using the Seahorse XF Analyzer. As shown in Figure 2A, OCR was drastically diminished in sperm from NKAα4 KO mice compared to WT sperm. The basal OCR of NKAα4 KO sperm was six-fold lower than that of WT sperm, indicative of minimal mitochondrial respiration (Figure 2B). Upon addition of FCCP, and the electron transport chain inhibitors, AA + R, WT sperm displayed a normal response, with first an increase and then a decrease in OCR respectively (Figures 2A,C,D). In contrast, sperm from NKAα4 KO mice displayed little or no response to these various pharmacological manipulations, due to reduced mitochondrial function (Figures 2A,C,D).

**FIGURE 3:**
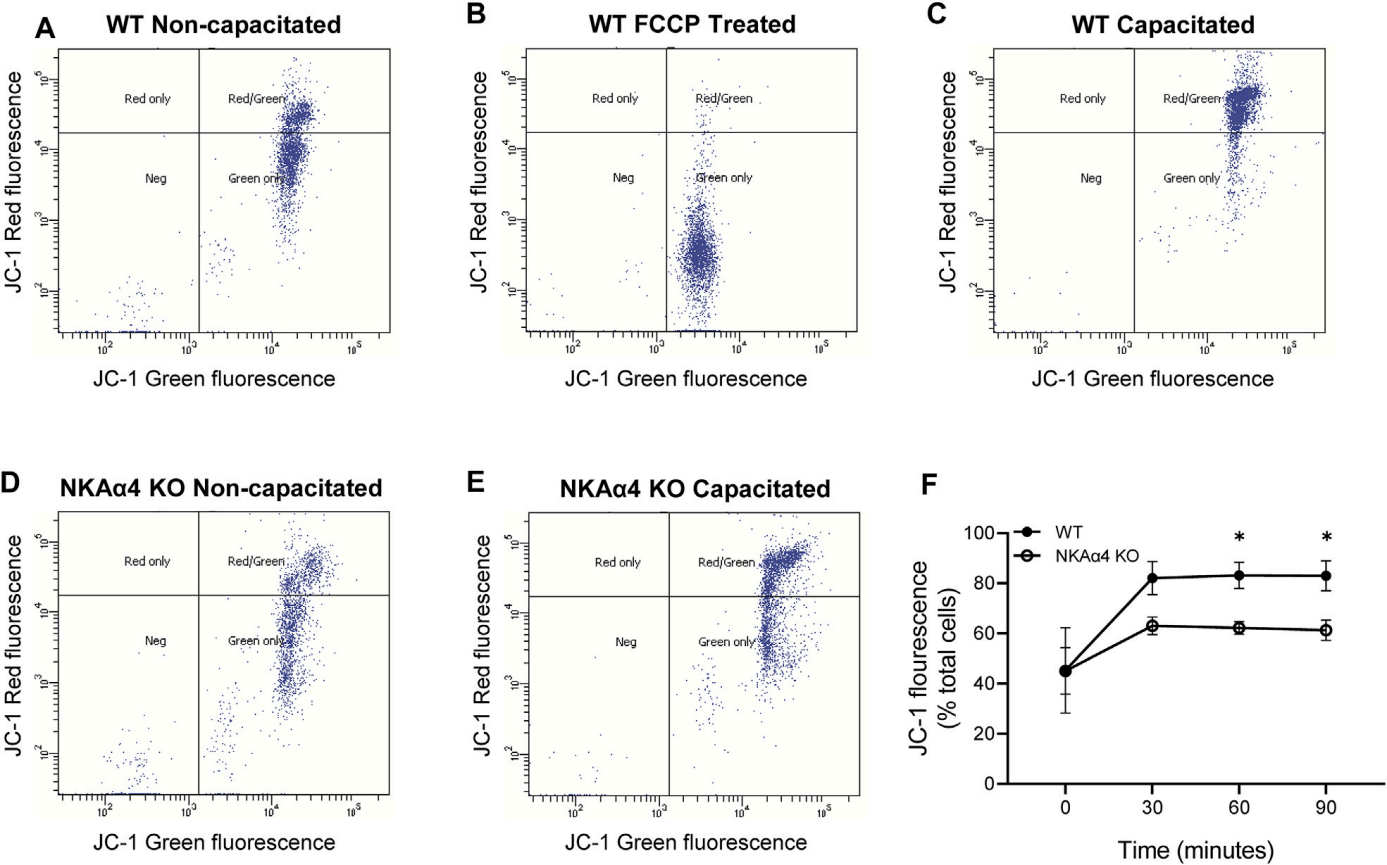
NKAα4 KO sperm display alterations in mitochondrial membrane potential. **(A–F)** Mitochondrial membrane potential in WT and NKAα4 KO sperm was determined by assessing the monomeric (green fluorescence) or aggregate (red fluorescence) forms of JC-1 using a flow cytometer. **(A)** WT sperm non-capacitated. **(B)** WT sperm non-capacitated treated with FCCP as a control for mitochondrial depolarization. **(C)** WT sperm capacitated for 90 min. **(D)** NKAα4-KO sperm non-capacitated. **(E)** NKAα4-KO sperm capacitated for 90 min. **(F)** Mitochondrial membrane potential in WT and NKAα4-KO sperm under non-capacitated conditions or after different times (30, 60, and 90 min) in capacitation medium. The data is represented as the percentage of cells with red/green fluorescence over the total number of cells. Experiments were performed in triplicates, with the asterisks representing a significance of ∗*p* < 0.05.

The impairment in oxidative phosphorylation suggests that mitochondrial function is disrupted in sperm from NKAα4 KO mice. To further study mitochondrial function, we measured mitochondrial membrane potential in WT and NKAα4 KO sperm under non-capacitated and capacitated conditions, using the potentiometric dye, JC-1, and flow cytometry. The JC-1 dye is a lipophilic agent that accumulates in mitochondria in a potential-dependent manner. In its natural monomeric form, JC-1 exhibits green fluorescence, but at higher potentials it forms aggregates that have an excitation and emission in the red spectrum. This change in fluorescence is an indicator of the transmembrane potential of mitochondria. When JC-1 was added to non-capacitated WT sperm, a significant number of cells showed the positive red and green fluorescence of healthy polarized mitochondria (Figure 3A). Treatment with the mitochondrial uncoupler FCCP resulted in a loss of cells with red/green fluorescence in favor of a green only pattern, typical of mitochondrial depolarization (Figure 3B). Capacitation of WT sperm led to an increase in the number of cells positive for red/green fluorescence, indicative of higher mitochondrial membrane potential (Figure 3C). When JC-1 was applied to NKAα4 KO sperm, the cells displayed a similar pattern of fluorescence as compared to non-capacitated WT sperm (Figure 3D). However, capacitated NKAα4 KO sperm did not exhibit the same shift in red/green fluorescence that was observed in capacitated WT sperm (Figure 3E). This demonstrates that sperm from NKAα4 KO mice do not display the normal capacitation-induced increase in mitochondrial membrane potential. The reduction in mitochondrial membrane potential was observed at various time points during capacitation, as summarized in Figure 3F. Altogether, these results demonstrate that NKAα4 is important for maintaining mitochondrial membrane potential during sperm capacitation.

### The Redox State of NKAα4 KO Sperm is Altered

**FIGURE 4:**
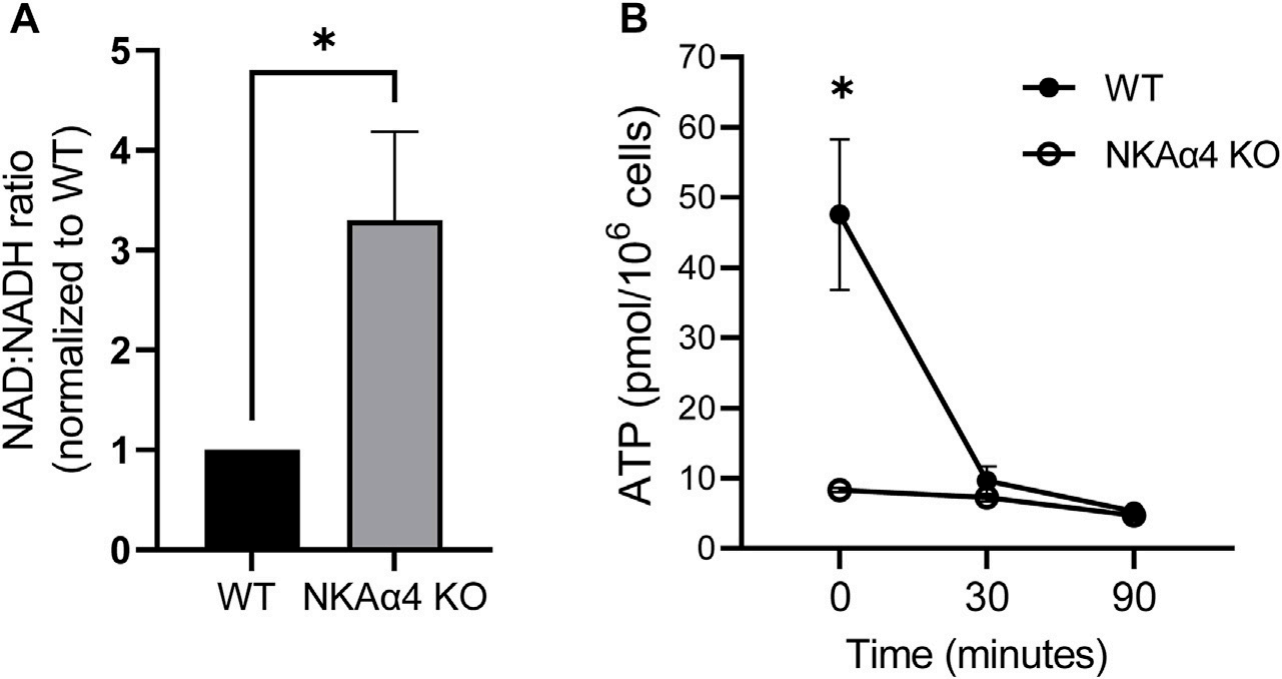
Sperm from WT and NKAα4 KO sperm display alterations in the NAD/NADH ratio and ATP levels. **(A)** The NAD/NADH ratio was measured in WT and NKAα4 KO sperm using a colorimetric kit. The graph represents the NAD/NADH ratio normalized to WT sperm. The data is expressed as the mean ± SEM, with an n = 5. Asterisks indicate a significance of ∗*p* < 0.05. **(B)** ATP levels were measured in capacitated WT (filled circles) and NKAα4 KO (open cirlces) sperm at 0, 30, and 90 min. ATP content was normalized to cell number (pmol/10^6^ cells). The data is expressed as the mean ± SEM of 3 separate experiments, with asterisks indicating significance of ∗*p* < 0.05.

The reduction in glycolytic activity and mitochondrial respiration in sperm from NKAα4 KO mice would be expected to impact the cellular redox state. We estimated the redox state of NKAα4 KO sperm by determining the ratio of the reduced and oxidized forms of nicotinamide adenine dinucleotide (NAD/NADH). As shown in Figure 4A, sperm in which NKAα4 was deleted exhibited a three-fold higher NAD/NADH ratio, reflecting a relatively lower reduction of NAD to NADH compared to WT sperm. These results further reinforce the idea that sperm lacking NKAα4 have impaired metabolic activity.

### NKAα4 KO Sperm Have Low ATP Levels

ATP is an essential nucleotide generated during glycolysis and oxidative phosphorylation and is critical for sperm function. We measured ATP levels under non-capacitating and capacitating conditions and found that this high energy nucleotide is significantly reduced in non-capacitated NKAα4 KO sperm compared to WT sperm (Figure 4B). In WT sperm, the levels of ATP were reduced upon capacitation, likely reflecting the high degree of ATP consumption that accompanies this process (Figure 4B, filled circles). Interestingly, ATP levels in the NKAα4 KO sperm remained consistently low regardless of their capacitated or non-capacitated state (Figure 4B, open circles).

### Sperm From NKAα4 KO Mice Display Oxidative Imbalance

**FIGURE 5:**
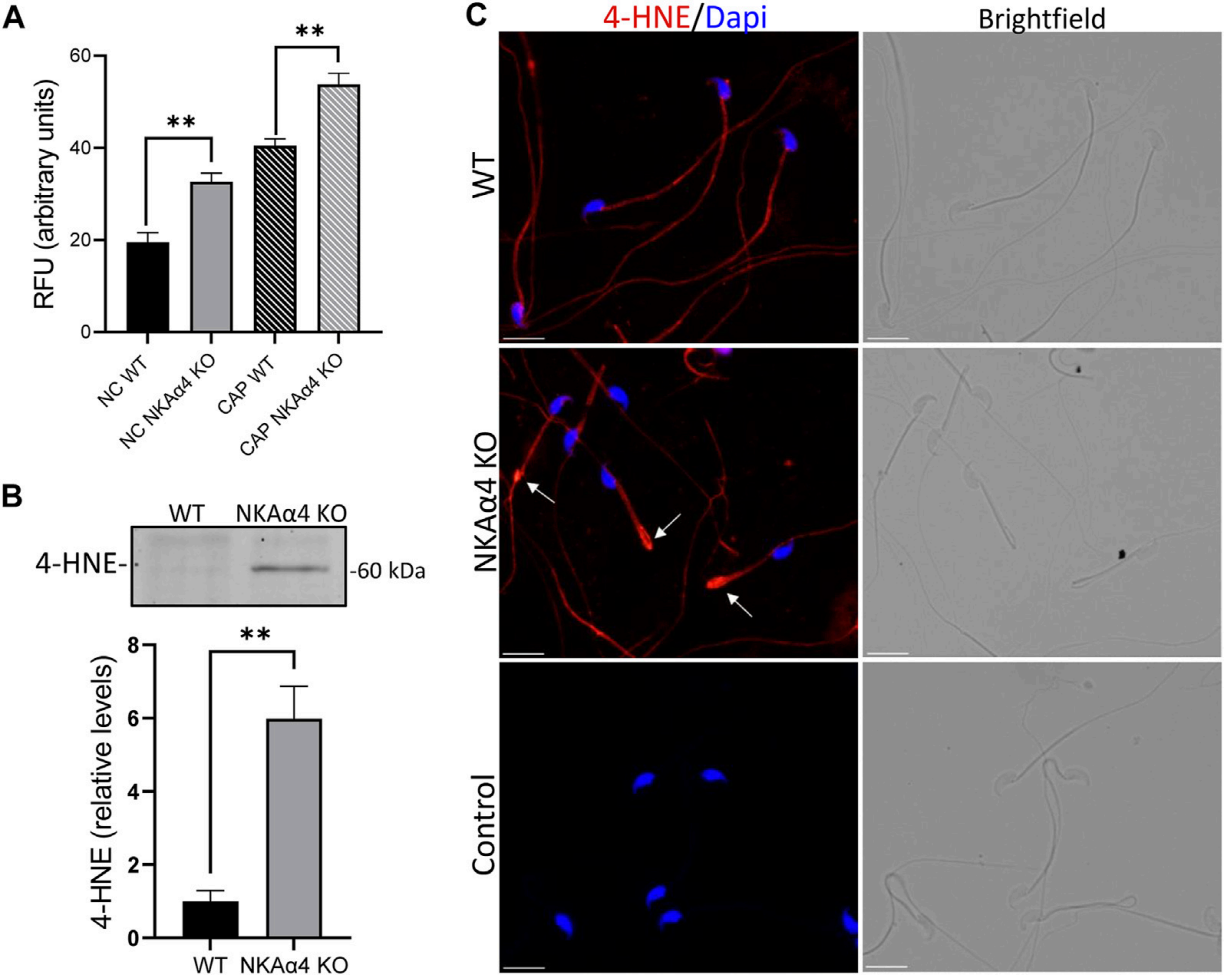
Reactive oxygen species and 4-HNE are elevated in NKAα4 KO sperm **(A)** Reactive oxygen species were measured in non-capacitated and capacitated sperm using the fluorescent probe, ROS Red Stain. The data is presented as the mean Relative Fluorescent Units (RFU) of 3–4 experiments. Significance is denoted by asterisks, with ∗∗*p* < 0.01. **(B)** 4-HNE was assessed in WT and NKAα4 KO sperm using an antibody generated against protein adducts of 4-HNE. Western blots were normalized to Coomassie staining, and quantified using ImageJ. The data is expressed as the relative levels of 4-HNE compared to WT sperm. **(C)** Representative images of 4-HNE staining in WT and NKAα4 KO sperm obtained using an Olympus IX-81 microscope. The white arrows indicate the sperm annulus. The scale bar represents 10 μM.

The cellular redox state is the result of a delicate balance between ROS levels produced during metabolism and the antioxidant protective systems that scavenge ROS. We determined the levels of ROS in sperm from NKAα4 KO mice under non-capacitating and capacitating conditions. Figure 5A demonstrates that ROS are significantly higher in NKAα4 KO sperm compared to WT sperm, under both non-capacitating and capacitating conditions. As expected, there was an increase in ROS in WT capacitated vs. non-capacitated sperm. However, capacitated NKAα4 KO sperm displayed an even greater increase in ROS than capacitated WT sperm.

Oxidants and free radicals can react with unsaturated fatty acids and lead to lipid peroxidation. A highly damaging product of lipid peroxidation is the electrophilic aldehyde, 4-hydroxynonenal (4-HNE), a common marker of cellular oxidative stress. As shown in Figure 5B, we utilized an antibody generated against 4-HNE modified proteins and found that NKAα4 KO sperm have higher amounts of 4-HNE than WT sperm. Immunocytochemical analysis showed 4-HNE staining mainly in the sperm flagellum and in higher levels at the junction between the midpiece and principal piece, as indicated by the white arrows (Figure 5C). Altogether, these results reveal that deletion of NKAα4 in sperm is accompanied by an increase in oxidative stress and lipid damage.

## Discussion

In this work, we made the unexpected observation that the testis-specific isoform of the NKA transporter plays an important role in sperm energetics. The effect of NKAα4 ablation has widespread consequences on energy production. This is reflected by the significant reduction in both glycolytic activity and oxidative phosphorylation in NKAα4 KO sperm.

The reduction of the ECAR in NKAα4 KO sperm is severe, reaching levels similar to those obtained by the addition of the glucose analog, 2-deoxyglucose. While mouse sperm can use a variety of substrates to support energy production, glycolysis plays a central role in sperm energetics, as shown by the functional alterations that follow the genetic deletion of several enzymes involved in glycolysis (Miki et al., 2004; Odet et al., 2008; Danshina et al., 2010; Nakamura et al., 2013; Ferramosca and Zara, 2014). This decrease in glycolytic activity could be due to a reduction in cytoplasmic glucose levels in NKAα4 KO sperm. Thus, previous results in our laboratory have shown that NKAα4 is the primary regulator of the transmembrane Na^+^ gradient in sperm and that impaired NKAα4 activity depresses glucose import via the sodium glucose transporter isoform 1 (SGLT-1) (Jimenez et al., 2011; Numata et al., 2022). Consequently, dissipation of the transmembrane Na^+^ gradient, caused by NKAα4 ablation, directly reduces glucose uptake. The decrease in glucose internalization would limit substrate availability to the glycolytic pathway, impairing the production of pyruvate and NADH. This in turn reduces mitochondrial energy production and shifts the NAD/NADH ratio. In support of this, the decreased glycolytic activity observed in NKAα4 KO sperm is accompanied by reduced mitochondrial respiration and alteration of the redox state, as demonstrated by a 3-fold increase in the NAD/NADH ratio.

Although glucose uptake in NKAα4 KO sperm is reduced, comprising approximately three-fourths of WT sperm glucose uptake (Numata et al., 2022), it is not completely suppressed. Therefore, other factors besides a decrease in glucose uptake may be contributing to the drastic reduction in sperm energetics caused by NKAα4 ablation. While it is unclear what additional mechanisms could be contributing to this phenotype, a link between Na,K-ATPase activity and glycolysis has also been demonstrated in somatic cells. Thus, inhibition of the Na,K-ATPase in cardiac and skeletal muscle cells leads to a decrease in glycolytic activity (James et al., 1999; Sepp et al., 2014). Na,K-ATPase activity appears to regulate phosphoglycerate kinase function in erythrocytes, affecting glycolytic activity in those cells (Mercer and Dunham, 1981). Moreover, increases in intracellular Na^+^ in myocardial cells affect mitochondrial NADH generation and the activity of the F_1_F_0_ ATPase (Murphy and Eisner, 2009; Liu et al., 2010); and [Na^+^]_i_ elevation has been associated with the mitochondrial malfunction that accompanies heart ischemia and failure (Murphy and Eisner, 2009; Hernansanz-Agustín and Enríquez, 2022). While additional experiments are planned to sort out the specific mechanisms that connect NKAα4 ion transport and mitochondrial function, our study is the first report of the link between energetics and Na,K-ATPase function in non-somatic cells.

Measurements of OCR supported the idea that NKAα4 activity is also linked to oxidative phosphorylation in sperm. Deletion of NKAα4 was accompanied by depolarization of the mitochondrial membrane potential, suggesting mitochondrial dysfunction. As mentioned above, this could be secondary to the reduction in glycolytic activity, which provides pyruvate and the acetyl groups that fuel the Krebs cycle and in turn, mitochondrial oxidative phosphorylation. Alternatively, maintenance of the transmembrane Na^+^ gradient by NKAα4 could affect mitochondrial function. The distribution of NKAα4 in the midpiece of the sperm flagellum, close to the underlying mitochondria, could facilitate this functional link between NKAα4 and mitochondrial activity. Mitochondrial membrane potential has been linked to sperm fitness in several species, with highly motile sperm displaying an increase in mitochondrial membrane potential compared to immotile sperm (Agnihotri et al., 2016; Barbagallo et al., 2020). Our results show that deletion of NKAα4 results in a decrease in the population of cells that undergo hyperpolarization of the mitochondrial membrane potential during capacitation. The inability of NKAα4 KO sperm mitochondria to hyperpolarize during capacitation may also contribute to the infertility observed in these knockout mice.

Sperm are metabolically flexible cells that can utilize glycolysis or oxidative phosphorylation to supply the ATP required for their functional needs (Goodson et al., 2012; Balbach et al., 2020). Since deletion of NKAα4 impairs both pathways, it is not surprising that sperm from NKAα4 KO mice have low ATP levels. Similar to what others have shown, we find a reduction in ATP levels in WT sperm that have undergone capacitation (Sansegundo et al., 2022). This is likely reflecting the increased energy expenditure that these cells undergo during hyperactivation. In contrast, sperm from NKAα4 KO mice display lower ATP levels compared to WT sperm, regardless of their functional state. With scarce amounts of ATP, sperm function would be difficult to maintain. ATP levels are also necessary for the function of Na,K-ATPase and a large proportion of ATP is hydrolyzed in cells via this active transporter (Milligan and McBride, 1985). Therefore, the reduction in ATP could depress the activity of NKAα1, the other Na,K-ATPase expressed in sperm. The additional downregulation of NKAα1 activity would further impair the transmembrane Na^+^ gradient and exacerbate the functional defects of the NKAα4 null sperm.

The metabolic changes observed in NKAα4 KO sperm were accompanied by an increase in reactive oxygen species. The increase in ROS production could be attributed to a decrease in glucose uptake and the production of NADPH *via* the pentose phosphate pathway. Moreover, mitochondrial dysfunction can lead to oxidative stress and the production of ROS. We found that lipid peroxidation, a common byproduct of oxidative stress, is also higher in sperm devoid of NKAα4. Spermatozoa are highly vulnerable to oxidative stress due to their limited antioxidant capacity. Additionally, the high number of unsaturated fatty acids in their plasma membrane makes them particularly susceptible to lipid peroxidation by reactive oxygen species. This leads to the formation of the highly reactive lipid aldehydes, MDA and 4-HNE, which have been reported to form adducts with several different proteins in sperm (Nowicka-Bauer and Nixon, 2020). We have previously shown that deletion of NKAα4 results in morphological defects of the sperm flagellum, which display a break at the junction between the midpiece and principal piece (McDermott et al., 2021). Interestingly, we observed an accumulation of 4-HNE at the breakage site in the NKAα4 KO sperm flagellum. While it is unclear if ROS damage and lipid peroxidation are direct causes of these morphological defects, studies have shown that structural axonemal proteins are common targets of 4-HNE damage. Coincidentally, we also observed the downregulation of several structural proteins in sperm from NKAα4 KO mice (McDermott et al., 2021). Thus, ROS production and lipid peroxidation could be additional mechanisms by which deletion of NKAα4 leads to male infertility.

In conclusion, our results show that there is an important link between NKAα4 and sperm energetics, establishing a new mechanism that is essential for sperm function and male fertility.

## Data Availability

The raw data supporting the conclusion of this article will be made available by the authors, without undue reservation.
